# Physician-related factors associated with unscheduled revisits to the emergency department and admission to the intensive care unit within 72 h

**DOI:** 10.1038/s41598-020-70021-1

**Published:** 2020-08-03

**Authors:** Chao-Sheng Chang, Kuo-Hsin Lee, Hung-Yuan Su, Cheuk-Kwan Sun, I-Ting Tsai, Ying-Ying Lo, Chih-Wei Hsu

**Affiliations:** 1 Department of Emergency Medicine, E-Da Hospital, I-Shou University, No. 1, Yi-Da Road, Jiao-Su Village, Yan-Chao District, Kaohsiung, 82445 Taiwan; 2 In-Service Master Programs of Healthcare Administration, Department of Healthcare Administration, I-Shou University, Kaohsiung, 82445 Taiwan; 3 School of Medicine for International Student, I-Shou University, No. 8, Yi-Da Road, Jiao-Su Village, Yan-Chao District, Kaohsiung, Taiwan; 4 School of Chinese Medicine for Post Baccalaureate, I-Shou University, Kaohsiung, Taiwan

**Keywords:** Health care, Medical research

## Abstract

Investigation of physician-related causes of unscheduled revisits to the emergency department (ED) within 72 h with subsequent admission to the intensive care unit (ICU) is an important parameter of emergency care quality. Between 2012 and 2017, medical records of all adult patients who visited the ED and returned within 72 h with subsequent ICU admission were retrospectively reviewed by three experienced emergency physicians. Study parameters were categorized into “input” (Patient characteristics), “throughput” (Time spent on first ED visit and seniority of emergency physicians, and “output” (Charlson Comorbidity Index). Of the 147 patients reviewed for the causes of ICU admission, 35 were physician-related (23.8%). Eight belonged to more urgent categories, whereas the majority (n = 27) were less urgent. Patients who spent less time on their first ED visits before discharge (< 2 h) were significantly associated with physician-related causes of ICU admission, whereas there was no significant difference in other “input,” “throughput,” and “output” parameters between the “physician-related” and “non-physician-related” groups. Short initial management time was associated with physician-related causes of ICU admission in patients with initial less urgent presentations, highlighting failure of the conventional triage system to identify potentially life-threatening conditions and possibility of misjudgement because of the patients' apparently minor initial presentations.

## Introduction

An unscheduled revisit to the emergency department (ED) within 72 h is an important parameter for the assessment of emergency medical care quality^1,2^. There are myriad factors affecting the rate of 72-h ED revisit, including patient demography, ethnical and cultural differences, epidemiology of diseases, and the accessibility to medical care^3^. For instance, children and the elderly have been reported to have higher rates of ED revisits compared with those of other demographic groups^1,4^. From the perspective of emergency healthcare quality improvement, a number of studies have focused on preventable medical errors^5–7^. A previous study has reported a rate of medical error-related 72-h ED revisit between 5 and 45%^8^. In addition to the lack of physician experience and absence of standard operation guidelines for diagnosis, other reasons include ineffective communication, staff shortage, and mistakes made by other healthcare personnel^9^. All such factors adversely affect the quality of health care^10^. Among all the medical error-related causes, up to 8.2%–41.2% were physician related^1,11^.

A previous study, which investigated patients with ED revisits within 72 h and ICU admissions, showed a mortality of up to 27%^14^. The causes of ED revisits with subsequent ICU admission can be divided into those of physician-related (i.e., inadequate treatment and diagnostic errors) and those categorized as non-physician-related (i.e., illness-related and patient-related)^14^. It has been demonstrated that medical quality is more likely to be reflected by the prevalence of physician-related than that of patient- or disease-related causes of revisits^14^. Moreover, the rate of intensive care unit (ICU) admission for patients with unscheduled ED revisit within 72 h, which was found to be related to medical errors, has been reported to be 0.04–0.07%^11–14^. Delayed admission to the ICU has been reported to induce poor outcomes^15^. However, the prevalence of physician-related causes leading to delayed ICU admission, which is an important factor for patient safety improvement, remains unknown. Therefore, the present study investigated physician-related causes of ED revisit within 72 h with subsequent ICU admission, focusing on factors (e.g., demographic characteristics of patients, Taiwan Triage and Acuity Scale [TTAS], and experience of physicians) contributing to physician misjudgment.

## Results

### Study population

**Figure 1:**
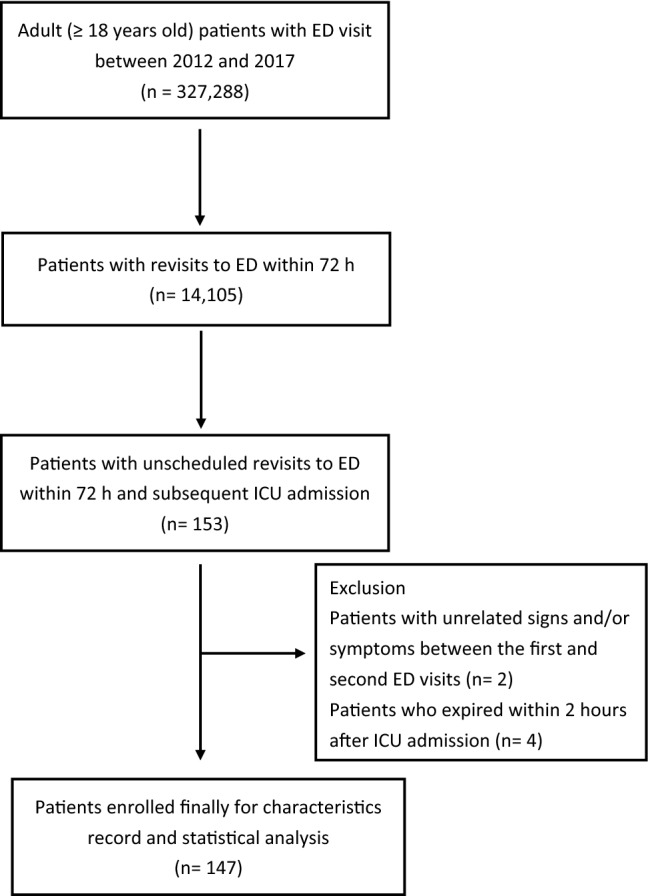
Flow chart of patient enrollment.

Of the 327,288 ED visits within the study period, 14,105 (4.31%) were revisits within 72 h. Of those patients revisiting the ED, 153 were admitted to the ICU (i.e., 0.047% of all ED visits). After excluding 6 patients who did not meet the inclusion criteria (Fig. 1), 147 patients in total were reviewed for the causes of ICU admission, which were physician related in 35 patients (23.8%) (i.e., 0.011% of all ED visits) and non-physician related in 112 patients (76.2%) (i.e., 0.034% of all ED visits) (Table 1).

**Table 1:** Study parameters and definitions.

Variable	Study parameters and definitions
**Input**	
Gender	Female and male
Age	Age ≥ 18 years
**Throughput**	
Chief complaints	The chief complaint is a concise statement describing the symptom, problem, and condition from patient Six categories: chest related, abdomen related, headache/consciousness related, fever, extremities related, and others
Urgency of disease presentation	TTAS^a^ (I, II), TTAS (III, IV, V)
Preliminary cause of ED visit	Trauma vs. non-trauma
Time of ED visit	Day (08:00–16:00), evening (16:00–24:00), and night (00:00–08:00) shifts
Time of first ED visit before discharge	< 2 vs. ≥ 2 h
Seniority of emergency physicians	< 3 vs. ≥ 3 years
**Output**	
System-based ICU diagnosis	Categories: respiratory/neurological/gastrointestinal/genitourinary/cardiovascular/other
Charlson Comorbidity Index (CCI)	CCI is a method of categorizing comorbidities of patients based on the International Classification of Diseases (ICD) diagnosis codes

^a^*TTAS* Taiwan Triage and Acuity Scale, a five-level scale for disease urgency assessment in the present study: Level I, resuscitation; Level II, emergency; Level III, urgent; Level IV, less urgent; and Level V, non-urgent.

### Association of input, throughput, and output factors with physician-related medical errors

The mean age of patients admitted to the ICU with physician-related causes was 62.40 ± 14.95 years, which was not significantly different from that of patients admitted to the ICU with non-physician-related causes (60.33 ± 15.91 years) (*p* = 0.579). Comparison of the genders between patients admitted to the ICU with physician-related and non-physician-related causes showed no significant difference between the two groups (*p* = 0.268) (Table 2). Therefore, the results excluded a significant impact of input factors on physician-related medical errors.

**Table 2:** The comparisons of physician-related and non-physical-related groups in three different categories of the conceptual model of saturation in emergencies.

Variable	Physician related		Non-physician related		Total	*p*-value
	N = 35 (23.8%) N (%)		N = 112 (76.2%) N (%)			
**Input**						
Age (median, IQR)	65 (57–71)		62.5 (49–73)			0.579^‡^
Gender						0.286^†^
Female	13	37.1	31	27.7	44	
Male	22	62.9	81	72.3	103	
**Throughput**						
Chief complaints						0.533^§^
1. Chest related	5	14.3	25	22.4	30	
2. Abdomen related	9	25.7	24	21.4	33	
3. Headache/consciousness related	9	25.7	23	20.5	32	
4. Fever related	5	14.3	13	11.6	18	
5. Extremities related	1	2.9	13	11.6	14	
6. Others	6	17.1	14	12.5	20	
Urgency of disease presentation						0.218^†^
1. TTAS I, II	8	22.9	38	33.9	46	
2. TTAS III, IV, V	27	77.1	74	66.1	101	
Preliminary cause of ED visit						0.067^†^
1. Non-trauma	27	77.1	100	89.3	127	
2. Trauma	8	22.9	12	10.7	20	
Time of ED visit						0.168^§^
1. Day	12	34.3	50	44.6	62	
2. Evening	16	45.7	32	28.6	48	
3. Night shifts	7	20.0	30	26.8	37	
Time of first ED visit before discharge						0.009^†^*
1. < 2 h	12	34.3	16	14.3	28	
2. ≥ 2 h	23	65.7	96	85.7	119	
Seniority of emergency physicians						0.578^†^
1. < 3 years	7	20.0	20	17.9	27	
2. ≥ 3 years	28	80.0	92	82.1	120	
**Output**						
System-based ICU diagnosis						0.674^§^
1. Respiratory	6	17.2	26	23.2	32	
2. Neurological	12	34.3	23	20.5	35	
3. Gastrointestinal	4	11.4	16	14.3	20	
4. Genitourinary	2	5.7	7	6.3	9	
5. Cardiovascular	9	25.7	35	31.2	44	
6. Other	2	5.7	5	4.5	7	
CCI (median, IQR)	3 (2.0–6.0)		4 (2.0–6.8)		0.236^‡^	

*TTAS* Taiwan Triage and Acuity Scale, *ED* emergency department, *ICU* intensive care unit, *CCI* Charlson Comorbidity Index.

Significance of difference determined using ^‡^Mann–Whitney test, ^†^Chi-squared test, and ^§^ANOVA; *p < 0.05.

Focusing on the throughput factors in both groups, the most common complaints (64.6%, 95/147) were abdomen, headache/consciousness, and chest pain related. The proportions of initial complaints related to physician-related causes of subsequent ICU admission were in the order of abdomen related (28.1%, 9/32), headache/consciousness related (27.3%, 9/33), fever related (27.8%, 5/18), chest pain related (16.7%, 5/30), and extremities related (7.1%, 1/14) (Table 2). When the urgency of disease on initial triage was considered, the majority of patients (68.7%, 101/147) belonged to the less urgent categories (i.e., Levels III and IV standing for urgent and less urgent, respectively). There were no patients belonging to Level V. Focusing on the 35 patients with physician-related causes of ICU admission, eight belonged to more urgent categories (i.e., Levels I and II), whereas the large majority (n = 27) fell into less urgent categories (i.e., Levels III and IV). In terms of significant difference between the two groups, patients who spent less time on their first ED visits before discharge (i.e., < 2 h) were more likely to be associated with physician-related causes of ICU admission than those having spent more time (i.e., ≥ 2 h) at the ED (42.9%, 12/28 vs. 19.3%, 23/119, respectively, *p* = 0.009). Although there is no significant difference in the preliminary cause of ED visit (i.e., trauma vs. non-trauma) between the two groups, patients with physician-related causes had a higher but statistically insignificant prevalence of trauma than that of the non-physician-related group (29.6% vs. 12%, *p* = 0.067) (Table 2). On the other hand, there was no significant difference between the two groups in other throughput factors, including the category of chief complaints, urgency of disease presentation (i.e., TTAS), time of ED visit (i.e., day, evening, and night shifts), and seniority of emergency physicians.

One of the output parameters was system-based ICU diagnosis. The most common diagnoses were in the order of cardiovascular (29.9%, 44/147), neurological (23.8%, 35/147), and respiratory (21.8%, 32/147) diseases. On the other hand, the probability associated with physician-related causes of ICU admission was in the order of neurological (34.3%, 12/35), cardiovascular (20.5%, 9/44), and respiratory (18.8%, 6/32) conditions. However, there was no significant difference between the two groups in this parameter (*p* = 0.674) (Table 2). When the other output parameter, Charlson Comorbidity Index (CCI), was considered, it was lower in physician-related causes of subsequent ICU admissions (i.e., 3.83 ± 2.65) compared with that in non-physician-related causes of subsequent ICU admissions (i.e., 4.32 ± 2.57), suggesting that physician-related causes of subsequent ICU admission were associated with patients with fewer comorbidities despite the lack of significant difference between the two groups (*p* = 0.236) (Table 2).

### Multiple logistic regression analysis

Multiple logistic regression analysis was performed for the identification of predictors for physician-related ICU admission among patients who revisited the ED within 72 h after their initial visits. Parameters included in the three sessions of ED patient management (i.e., input, throughput, and output) were set as independent variables. The results demonstrated significant association of physician-related causes with less time (i.e., < 2 h) being spent on the patients' first ED visits before discharge (*p* = 0.005) with an odds ratio of 4.046 (Table 3).

**Table 3:** Multiple logistic regression model using time of first ED visit before discharge.

Variables	Coefficient	Odds ratio	CI of 95%	*p*-value
**Age**				
≤ 65	1.110	3.033	0.748–12.305	0.12
> 65	–			
**Gender**				
Female	− 0.251	0.778	0.269–2.249	0.78
Male	–			
**Chief complaints**				
Chest-related	1.585	4.878	0.706–33.724	0.11
Abdomen-related	0.713	2.041	0.314–13.252	0.46
Headache/consciousness-related	0.502	1.651	0.326–8.362	0.54
Fever	− 0.084	0.919	0.166–5.092	0.92
Extremities-related	3.369	29.036	1.324–636.862	0.03
Others	–			
**Urgency of disease presentation**				
TTAS I, II	0.256	1.292	0.396–4.214	0.67
TTAS III, IV	–			
**Preliminary cause of ED visit**				
Non-trauma	0.743	2.103	0.456–9.694	0.34
Trauma	–			
**Time of ED visit**				
Day	− 0.014	0.986	0.264–3.687	0.98
Evening	− 0.158	0.854	0.253–2.888	0.80
Night shifts	–			
**Time of first ED visit before discharge**				
≤ 2 h	1.398	4.046	1.206–13.568	0.02*
> 2 h	–			
**Seniority of emergency physicians**				
≥ 3 years	0.289	1.335	0.395–4.519	0.64
< 3 years	–			
**System-based ICU diagnosis**				
Respiratory	0.162	1.176	0.077–18.023	0.91
Cardiovascular	− 0.140	0.87	0.061–12.425	0.92
Gastrointestinal	− 1.139	0.32	0.014–7.190	0.47
Genitourinary	− 0.745	0.475	0.017–13.143	0.66
**Neurological**	0.053	1.055	0.074–14.952	0.97
Other	–			
**CCI**	− 0.110	0.896	0.727–1.104	0.30

Hosmer–Lemeshow test = 3.270, p = 0.916.

*TTAS* Taiwan Triage and Acuity Scale, *ED* emergency department, *ICU* intensive care unit, *CCI* Charlson Comorbidity Index, *SD* standard deviation.

*p < 0.05.

### Characteristics of patients with subsequent ICU admission due to physician-related causes with time of first ED visit less than two hours

The characteristics of the 15 patients with ED revisits within 72 h and ICU admission due to physician-related causes with time of first ED visit less than two hours are summarized in Table 4. There was a male predominance (60%) with relatively large proportions of patients presenting with abdomen and headache/consciousness-related chief-complaints. The majority of patients did not initially manifest with urgent diseases (TTAS III/IV) and most belonged to the non-trauma category. In addition, only a minority (less than 7%) of patients visited the ED during night shifts. Regarding system-based diagnoses on admission to the ICU, neurological, cardiovascular, and gastrointestinal were the most common.

**Table 4:** Characteristics of patients with emergency department (ED) revisits within 72 h and intensive care unit (ICU) admission due to physician-related causes with time of first ED visit less than two hours (n = 15).

Parameter	n	%
**Gender**		
Female	6	40.0
Male	9	60.0
**Age (median, IQR)**		
(68, 61.0–71.0)		
**Chief complaints**		
Chest-related	2	13.3
Abdomen-related	3	20.0
Headache/consciousness-related	3	20.0
Fever	1	6.7
Extremities-related	2	13.3
Others	5	33.3
**Urgency of disease presentation**		
TTAS (I/II)	2	13.3
TTAS (III/IV/V)	13	86.7
**Preliminary cause of ED visit**		
Trauma	4	26.7
Non-trauma	11	73.3
**Time of ED visit**		
Day	8	53.3
Evening	6	40.0
Night shifts	1	6.7
**System-based ICU diagnosis**		
Respiratory	0	0.0
Neurological	5	33.3
Gastrointestinal	3	20.0
Genitourinary	1	6.7
Cardiovascular	4	26.7
Other	2	13.3
**CCI (median, IQR)**		
(4, 3.0–5.0)		

*TTAS* Taiwan Triage and Acuity Scale, *ED* emergency department.

## Discussion

Although the issue of ICU admission on ED revisits has been previously addressed, previous studies merely focused on the incidence^3^, mortality^16^, causes (i.e., physician, patient, and disease)^11,17^, and subjective analysis of medical errors from a patient's perspective^18^. Objective assessment of factors contributing to physician-related causes, however, has not been reported. The current study, which investigated the impact of the parameters in the three categories according to the conceptual model of saturation in emergencies (i.e., “input,” “throughput,” and “output”)^19^ on physician-related factors associated with patients' admission to the ICU during their revisits within 72 h, has several striking clinical implications. First, contrary to popular belief, less than one-third of those subsequently admitted to the ICU presented with urgent diseases/injuries (i.e., Levels I and II) on initial triage at the ED. Second, patients who spent less time on their first ED visits before discharge (i.e., < 2 h) were more likely to be associated with physician-related causes of subsequent ICU admission than those who spent more time (i.e., ≥ 2 h) during their initial ED visits. On the other hand, other parameters, namely, age and gender of patients, chief complaints, urgency of disease presentation, initial cause of ED visit, time of ED visit, seniority of emergency physicians, ICU diagnosis, and CCI, were not significant contributors to physician-related causes of ICU admissions.

The incidence of ICU admission on ED revisits in the current study was 0.046%, which was comparable with that in previous reports (i.e., 0.04–0.07%)^11–14^. Albeit rare (i.e., 0.011% of all ED visits in the current study), physician-related causes of delayed ICU admission are a major target for patient safety and medical quality improvement. The current study showed that the most common chief complaints related to physician-related causes were headache/consciousness or abdomen related, highlighting possible Achilles heels of the current triage system that fail to objectively assess urgency of some diseases when the patient presents with apparently stable vital signs and a relatively low pain score. This was also supported by the finding that the majority of these patients (77.2%) were categorized into the non-emergent categories (i.e., Levels III and IV) during the initial triage. These findings underscored the importance of physician discretion regarding the need for further evaluation in patients presenting with apparently stable vital signs but equivocal physical findings. In the current study, headache/consciousness-related complaints were most likely associated with physician-related causes of delayed ICU admission (n = 12, 34.3%). Although sudden-onset severe vertigo may be a benign presentation (e.g., benign paroxysmal positional vertigo), it could be a manifestation of subarachnoid hemorrhage when accompanied by progressive tension headache, migraine, or nonspecific neurological symptoms^20^. Besides, signs and symptoms of cerebellar hemorrhage or infarction involving impaired coordination and unsteady gait are likely to be missed at the ED^21^. Indeed, a previous study has demonstrated that up to 6% of patients may subsequently experience severe or fatal neurological complications even after neurologist consultation in the emergency setting^22^. Other contributing factors may be a lack of neurological diagnostic training among emergency physicians, underestimation of urgency of patient's condition, or failure to order appropriate imaging studies for patients with equivocal diagnosis.

Incorporating the concept of the conceptual model of saturation in emergencies^19^ and the factors reported to affect the prognosis of patients in the ICU^23^, the current study investigated 10 parameters that may be associated with physician-related causes of ICU admission during patient revisits (Table 2). In the present study, the finding of a significant association between a shortened stay of initial ED visits (< 2 h) (i.e., one of the “throughput” parameters) and physician-related causes of ICU admission (i.e., an odds ratio of 4.046) may partly be explained by the relatively non-urgent presentations in the majority of patients. The result was not surprising because emergency physicians tend to discharge patients with apparently minor conditions to avoid ED overcrowding, which is known to adversely affect patient satisfaction and patient prognosis^24^. On the other hand, other parameters, namely, age and gender of patients, chief complaints, urgency of disease presentation, initial cause of ED visit, time of ED visit, seniority of emergency physicians, ICU diagnosis, and CCI, were not significant contributors to physician-related causes of ICU admissions. The findings may be explained by an increased level of physician alertness on encountering patients with advanced age, those with more urgent presentation on triage, and those with comorbidities. The lack of significance of physician seniority as a significant contributor may be attributed to the fact that only attending physicians were involved and residents were excluded from the present study.

Compared with the CTAS, the TTAS also takes into account changes in vital signs, consciousness level, pain severity, and mechanism of trauma^25^. Nevertheless, the findings of the present study showed that the currently used five-class Taiwanese triage system focuses on the stability of vital signs, which may not show notable changes in life-threatening conditions such as myocardial ischemia, subarachnoid hemorrhage, and cerebellar ischemia. The results, therefore, suggest that a high degree of vigilance among members of the emergency care team is needed in the management of patients presenting apparently relatively minor symptoms, especially neurological symptoms. A previous meta-analytic study demonstrated that most commonly used triage systems, such as CTAS, ESI and MTS, demonstrated a moderate to high validity to identify high- and low-urgency patients. Nevertheless, their performance was found to be highly variable due to differences in study design and populations as well as reference standards that precluded a valid comparison^26^. Therefore, the superiority of other systems to TTAS in detecting aspects noted to be missing from the TTAS remains unclear.

One of the output parameters was system-based ICU diagnosis. The most common diagnoses were in the order of cardiovascular (29.9%, 44/147), neurological (23.8%, 35/147), and respiratory (21.8%, 32/147) conditions. On the other hand, the probability associated with physician-related causes of ICU admission was in the order of neurological (34.3%, 12/35), cardiovascular (20.5%, 9/44), and respiratory (18.8%, 6/32) conditions. However, there was no significant difference between the two groups in this parameter (*p* = 0.674) (Table 2). When the other output parameter, CCI, was considered, it was lower in physician-related causes (i.e., 3.83 ± 2.65) compared with that in non-physician-related causes (i.e., 4.32 ± 2.57), suggesting that physician-related causes of subsequent ICU admission was associated with patients with fewer comorbidities despite the lack of significant difference between the two groups (*p* = 0.236) (Table 2).

The results of the current study demonstrated that a management time < 2 h during the first ED visit was significantly associated with physician-related causes of ICU admission on the patients' second visit. The result was consistent with the finding of non-emergent category in the majority of patients on their initial visits, which may contribute to physicians' underestimation of disease urgency and failure to order more detailed examinations. Besides, although cardiovascular diseases were the most common cause of ICU admission, physician-related causes were most likely associated with neurological conditions. The findings not only underscored the failure of the conventional triage system to initially identify potentially life-threatening conditions but also highlighted the possibility of misjudgment because of the patients' apparently minor initial presentations. Our study suggested the need for a high degree of vigilance and the importance of physician discretion in the management of patients with apparently minor presentations. The results of the current study may suggest the benefit of regular re-triaging after primary survey as well as the need for inter-professional communication for equivocal cases. In addition, post-discharge instructions for the patients and their family may help in early recognition of the deterioration of the patients’ condition and prevention of subsequent ICU admissions.

The present study had its limitations. First, because the findings were from a single tertiary referral center with a limited number of eligible patients despite a study period of 6 years, the results may not be statistically sound and suitable for being extrapolated to other clinical settings. Second, although three supposedly unbiased experts were responsible for assigning the cases to physician-related and non-physician-related causes, different opinions may exist, and a final decision was made according to the majority of reviewers. Therefore, albeit unlikely, the possibility of bias still existed.

## Methods

### Study setting and population

Between January 2012 and December 2017, the medical records of all adult patients (age ≥ 18 years) who visited the ED of a single tertiary referral center and returned to the ED within 72 h after the first visit with subsequent ICU admission were retrospectively reviewed by three experienced emergency physicians. Patients with (1) unrelated signs and/or symptoms between the first and second ED visits, and (2) an ICU stay < 24 h were excluded from the study. All patients were then reviewed and divided into those with subsequent ICU admission due to physician-related and non-physician-related causes; the physician-related causes were defined as patients being deemed suitable for discharge due to incomplete/incorrect diagnosis and/or treatment during the patient's first visit. This observational study was approved by the Institutional Review Board of E-Da hospital (No. EMRP-107-065), and the requirement of informed consent of patients was waived owing to the retrospective observational nature of the study. All methods were performed in accordance with the relevant guidelines and regulations.

### Determination of physician-related causes

One important issue is the objective determination of whether the cause of ED revisit with ICU admission is medical error related; most previous studies retrospectively categorized the causes in accordance with the opinions of a group of experts^13^. However, human factors may interfere with the categorization depending on the expertise of the group members. For instance, the indication for prescription of medication or examination may vary among physicians of different specialties^27^. The present study enrolled three physicians with over 10 years' experience of emergency medical practice (i.e., at least 10 years after board certification) as reviewers for determining the cause. All three physicians were not involved in the management of all the cases to be discussed. The cause was determined when the majority (i.e., two or more) of the reviewers agreed on a decision.

### Study parameters and definitions

The current study was designed on the basis of the conceptual model of saturation in emergencies^19^. In brief, the parameters were categorized into “input” (i.e., patient source and characteristics) including age and gender; “throughput” (i.e., management procedures at the ED) including body part-based chief complaints (i.e., six categories: chest related, abdomen related, headache/consciousness related, fever, extremities related, and others)^28–31^, urgency of disease presentation (i.e., TTAS), preliminary cause of ED visit (trauma vs. non-trauma), time of ED visit (i.e., day (08:00–16:00), evening (16:00–24:00), and night (00:00–08:00) shifts), time that the patient spent on the first ED visit before discharge (i.e., < 2 vs. ≥ 2 h)^10,32^, and seniority of emergency physicians (i.e., < 3 vs. ≥ 3 years; and “output,” namely, system-based ICU diagnosis and Charlson Comorbidity Index (CCI^11–14,23^ (Table 1). A cut-off point of 2 h was chosen for assessing the adequacy of assessment and management on the patients' initial ED visits based on the reported finding of a duration of 2 h for an average patient visit at the ED in Taiwan^33^. TTAS, which was a five-level scale used for disease urgency assessment in the present study, is a modification of the Canadian Triage and Acuity Scale (CTAS),besides chief complaints, TTAS also takes into account vital signs, consciousness level, pain severity, and mechanism of trauma. There are five levels of urgency: Level I, resuscitation; Level II, emergency; Level III, urgent; Level IV, less urgent; and Level V, non-urgent^25,26^. The triage nurses certified after two years of training by the Taiwan Society of Emergency Medicine are responsible for the conduction of the triage process. In our clinical practice, trauma is defined as a physical injury caused by an external force of violence^34^, whereas the rest of the ED visits due to other causes belong to the non-trauma category.

### Statistical analysis

All data were analysed using SPSS version 22 (IBM Corp., Armonk, N.Y., USA, https://www.ibm.com/products/spss-statistics). Descriptive statistics was performed to assess the associations between the study parameters and physician-related causes. Significance of difference between categorical variables was determined using chi-squared test, whereas that between continuous variables was assessed using paired t-test and Mann–Whitney test for those with and without normal distribution, respectively. Univariate analysis was adopted to identify potential factors related to physician-related medical errors. Logistic regression analysis was performed to pinpoint independent predictor(s) of medical errors. A probability value (*p*) < 0.05 is considered statistically significant.

## Data Availability

Data relevant to the present study are available on request made to the corresponding author.

## References

[1] Nunez S, Hexdall A, Aguirre-Jaime A (2006). Unscheduled returns to the emergency department: an outcome of medical errors?. BMJ Qual. Saf..

[2] Sørup CM, Jacobsen P, Forberg JL (2013). Evaluation of emergency department performance: a systematic review on recommended performance and quality-in-care measures. SJTREM..

[3] Han CY (2015). Early revisit to the emergency department: an integrative review. J. Emerg. Nurs..

[4] Huang YC, Cheung SC, Yang E, Lu A (2010). Analysis of early discharge of admitted emergency department boarders. J. Emerg. Med. Taiwain..

[5] Benbassat J, Taragin M (2000). Hospital readmissions as a measure of quality of health care: advantages and limitations. Arch. Intern. Med..

[6] Chan AHS (2016). Characteristics of patients who made a return visit within 72 hours to the emergency department of a Singapore tertiary hospital. SMJ..

[7] Dhingra KR, Elms A, Hobgood C (2010). Reducing error in the emergency department: a call for standardization of the sign-out process. Ann. Emerg. Med..

[8] Trivedy CR, Cooke MW (2015). Unscheduled return visits (URV) in adults to the emergency department (ED): a rapid evidence assessment policy review. J. Emerg. Med..

[9] Moskop JC, Geiderman JM, Hobgood CD, Larkin GL (2006). Emergency physicians and disclosure of medical errors. Ann. Emerg. Med..

[10] Rodziewicz, T. L. & Hipskind, J. E. Medical error prevention. In StatPearls [Internet]: StatPearls Publishing (2019). 29763131

[11] Wu CL (2010). Unplanned emergency department revisits within 72 hours to a secondary teaching referral hospital in Taiwan. J. Emerg. Med..

[12] Fan, J. S. *et al*. Risk factors and prognostic predictors of unexpected intensive care unit admission within 3 days after ED discharge. *Am. J. Emerg. Med.* **25**, 1009–1014 (2007). 10.1016/j.ajem.2007.03.005 18022494

[13] Hu KW, Lu YH, Lin HJ, Guo HR, Foo NP (2012). Unscheduled return visits with and without admission post emergency department discharge. J. Emerg. Med..

[14] Tsai IT (2016). Characteristics and outcomes of patients with emergency department revisits within 72 hours and subsequent admission to the intensive care unit. TCMJ..

[15] Cardoso LT (2011). Impact of delayed admission to intensive care units on mortality of critically ill patients: a cohort study. Crit. Care..

[16] Cheng, S. Y. *et al*. The characteristics and prognostic predictors of unplanned hospital admission within 72 hours after ED discharge. *Am. J. Emerg. Med.* **31**, 1490–1494 (2013). 10.1016/j.ajem.2013.08.004 24029494

[17] Croskerry P, Sinclair D (2001). Emergency medicine: a practice prone to error?. CJEM..

[18] Kooienga S, Stewart VT (2011). Putting a face on medical errors: a patient perspective. J. Healthc. Qual..

[19] Asplin BR (2003). A conceptual model of emergency department crowding. Ann. Emerg. Med..

[20] Kowalski RG (2004). Initial misdiagnosis and outcome after subarachnoid hemorrhage. JAMA.

[21] Sangha N (2014). Misdiagnosis of cerebellar infarctions. Can. J. Neurol. Sci..

[22] Royl G, Ploner CJ, Leithner C (2011). Dizziness in the emergency room: diagnoses and misdiagnoses. Eur. Neurol..

[23] Unal AU (2015). Prognosis of patients in a medical intensive care unit. North. Clin. Istanb..

[24] Yarmohammadian, M. H., Rezaei, F., Haghshenas, A. & Tavakoli, N. Overcrowding in emergency departments: a review of strategies to decrease future challenges. *J. Res. Med. Sci. 22*, (2017). 10.4103/1735-1995.200277 PMC5377968 28413420

[25] Ng CJ (2010). Comparison between Canadian triage and acuity scale and Taiwan triage system in emergency departments. J. Formos. Med. Assoc..

[26] Zachariasse JM (2019). Performance of triage systems in emergency care: a systematic review and meta-analysis. BMJ Open..

[27] Mosadeghrad AM (2014). Factors affecting medical service quality. Iran J. Public Health..

[28] Berman P, Hogan D, Fox RA (1987). The atypical presentation of infection in old age. Age Ageing..

[29] Khafaji HA, Al Suwaidi JM (2014). Atypical presentation of acute and chronic coronary artery disease in diabetics. WJC..

[30] Richter JE (1996). Typical and atypical presentations of gastroesophageal reflux disease: the role of esophageal testing in diagnosis and management. Gastroenterol. Clin..

[31] Waterer GW, Kessler LA, Wunderink RG (2006). Delayed administration of antibiotics and atypical presentation in community-acquired pneumonia. Chest.

[32] Yoon P, Steiner I, Reinhardt G (2003). Analysis of factors influencing length of stay in the emergency department. Can. J. Emerg. Med..

[33] Chaou CH (2017). Predicting length of stay among patients discharged from the emergency department—using an accelerated failure time model. PLoS ONE.

[34] Hokkam E (2015). Trauma patterns in patients attending the Emergency Department of Jazan General Hospital Saudi Arabia. World J. Emerg. Med..

